# Sorafenib Modulates the LPS- and Aβ-Induced Neuroinflammatory Response in Cells, Wild-Type Mice, and 5xFAD Mice

**DOI:** 10.3389/fimmu.2021.684344

**Published:** 2021-05-27

**Authors:** Jieun Kim, Jin-Hee Park, Seon Kyeong Park, Hyang-Sook Hoe

**Affiliations:** ^1^ Department of Neural Development and Disease, Korea Brain Research Institute (KBRI), Daegu, South Korea; ^2^ Department of Brain & Cognitive Sciences, Daegu Gyeongbuk Institute of Science & Technology (DGIST), Daegu, South Korea

**Keywords:** LPS, NF-kB, STAT3, Sorafenib, AKT, Microglia

## Abstract

Sorafenib is FDA-approved for the treatment of primary kidney or liver cancer, but its ability to inhibit many types of kinases suggests it may have potential for treating other diseases. Here, the effects of sorafenib on neuroinflammatory responses *in vitro* and *in vivo* and the underlying mechanisms were assessed. Sorafenib reduced the induction of mRNA levels of the proinflammatory cytokines COX-2 and IL-1β by LPS in BV2 microglial cells, but in primary astrocytes, only COX-2 mRNA levels were altered by sorafenib. Interestingly, sorafenib altered the LPS-mediated neuroinflammatory response in BV2 microglial cells by modulating AKT/P38-linked STAT3/NF-kB signaling pathways. In LPS-stimulated wild-type mice, sorafenib administration suppressed microglial/astroglial kinetics and morphological changes and COX-2 mRNA levels by decreasing AKT phosphorylation in the brain. In 5xFAD mice (an Alzheimer’s disease model), sorafenib treatment daily for 3 days significantly reduced astrogliosis but not microgliosis. Thus, sorafenib may have therapeutic potential for suppressing neuroinflammatory responses in the brain.

## Introduction

Neuroinflammation protects nervous tissue in the central nervous system (CNS) in response to a variety of cues, including infection, traumatic brain injury, toxic metabolites, or autoimmunity (1). In this process, microglia and astrocytes act as first responders (2). Microglia actively survey various cues of the environment and significantly change their morphology in response to neural injury (3). Activated microglia communicate with neighboring neurons and/or other glial cells, leading to the activation of and morphological changes in astrocytes, the most abundant cell type in the brain and supporters of neurons (4). Activated microglia and astrocytes release various proinflammatory cytokines in the brain, including COX-2, IL-1β, IL-6 and iNOS (2), which is the first step in intensifying neuroinflammation in the CNS. Therefore, the identification of therapeutic molecular targets in the neuroinflammatory response would facilitate the development of drugs to prevent/treat neuroinflammation-associated diseases.

Lipopolysaccharide (LPS) is an endotoxin that strongly activates the neuroinflammatory response in the CNS. LPS, an outer membrane component of gram-negative bacteria, binds Toll-like receptors (TLRs) in several cell types, most notably dendritic cells, microglia and astrocytes (5). As a TLR ligand, LPS activates downstream signaling pathways of TLRs, including mitogen-activated protein kinase (MAP) kinase and protein kinase B (AKT) signaling and/or the transcription factors signal transducer and activator of transcription 3 (STAT3) and nuclear factor kappa-light-chain-enhancer of activated B cells (NF-kB). In turn, the activation of these pathways initiates proinflammatory cytokine release and neuroinflammation in glial cells. Emerging evidence indicates that proinflammatory cytokine release by glial cells is a crucial marker of neuroinflammation (6–8). Thus, inhibiting the LPS-evoked neuroinflammatory response may prevent neuroinflammation.

Sorafenib, an anti-cancer drug used in the treatment of kidney and liver cancer, inhibits several kinases, including vascular endothelial growth factor receptor (VEGFR) kinases, platelet-derived growth factor receptor (PDGFR) kinases, and rapidly accelerated fibrosarcoma (RAF) kinases (9, 10). Sorafenib also decreases MAP kinase signaling (i.e., ERK, JNK, and p38), resulting in suppression of tumor growth in lymphoma xenograft mice and cell death of thyroid carcinoma cells (11–13). In addition, sorafenib reduces STAT3-associated IL-6 and NF-kB-linked COX-2 levels in hepatocellular carcinoma cells and APPswe mice, respectively (14–16). Sorafenib crosses the blood-brain barrier (BBB) (13, 17), but whether sorafenib modulates glial activation (microgliosis and astrogliosis) as well as LPS-induced neuroinflammation in glia-specific cell lines, wild-type mice, and 5xFAD mice has not been comprehensively investigated.

Here, we show that sorafenib reduces the induction of COX-2 and IL-1β mRNA expression by LPS in BV2 microglial cells. In primary astrocytes, sorafenib diminishes the increase in COX-2 mRNA levels induced by LPS but has no effect on other proinflammatory cytokines modulated by LPS treatment. Sorafenib also suppresses the LPS-induced increases in STAT3 and NF-kB phosphorylation levels in BV2 cells by inhibiting AKT and P38 signaling. In addition, in LPS-injected wild-type mice, sorafenib treatment significantly decreases microgliosis- and astrogliosis-linked COX-2 levels and, consistent with the effects observed in BV2 cells, reduces AKT phosphorylation. Moreover, sorafenib administration daily for 3 days significantly reduces Aβ-induced astrogliosis but not microgliosis in 5xFAD mice. Thus, the anti-cancer drug sorafenib modulates LPS-induced glial activation and neuroinflammatory responses both *in vitro* and *in vivo*.

## Materials and Methods

### Ethics Statement

All experiments were approved by the institutional biosafety committee (IBC) and performed in accordance with approved animal protocols of the Korea Brain Research Institute (KBRI, approval no. IACUC-19-00042).

### Sorafenib

**Figure 1:**
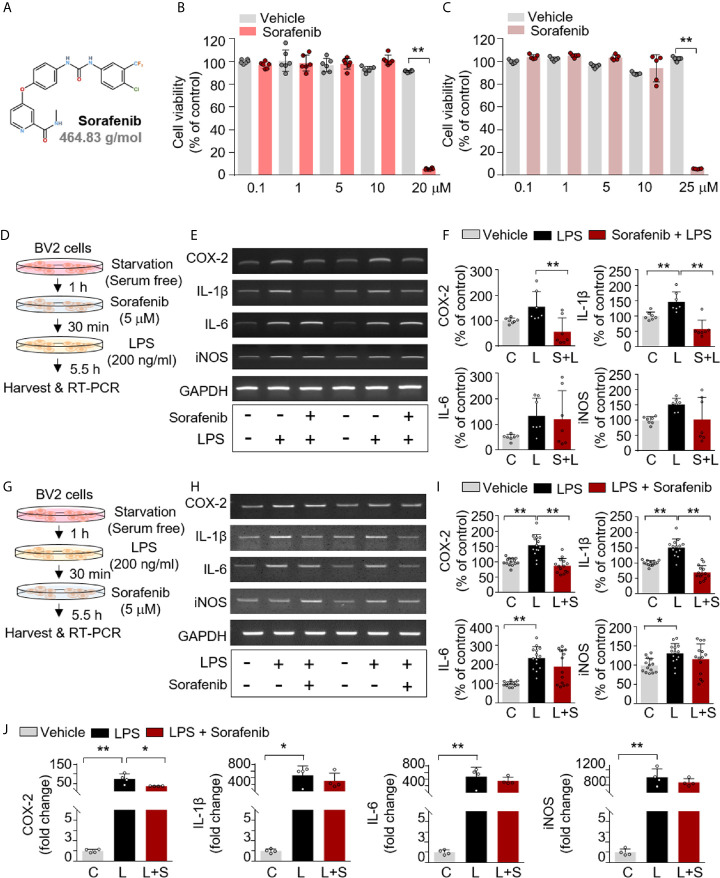
Sorafenib decreases LPS-induced proinflammatory cytokine levels *in vitro*. **(A)** Structure of sorafenib. **(B, C)** The cytotoxicity of sorafenib due to mitochondrial arrest was assessed by the MTT assay in BV2 microglial cells treated for 6 or 24 h with a range of concentrations (0.1, 1, 5, 10, and 20 or 25 μM) or vehicle (1% DMSO) (6 h, n= 6/dose; 24 h, n= 5/dose). **(D)** Scheme for pre-treatment of BV2 cells with sorafenib. **(E, F)** RT-PCR analysis of proinflammatory cytokine levels in BV2 cells treated as described in **(D)** (n=7/group). **(G)** Scheme for post-treatment of BV2 cells with sorafenib. **(H, I)** RT-PCR analysis of proinflammatory cytokine levels in BV2 cells treated as described in **(G)** (n= 14/group). **(J)** Real-time PCR analysis of proinflammatory cytokine levels in cultured primary astrocytes (n= 4/group). *p < 0.05, **p < 0.01.

Sorafenib was purchased from Cayman Chemical (Ann Arbor, MI, USA; Cat. No. 10009644) (**Figure 1A**). Based on the results of MTT assays, a sorafenib concentration of 5 μM (in DMSO) was used for cell experiments. For animal experiments, sorafenib was intraperitoneally (i.p.) administered at 10 mg/kg dissolved in 5% DMSO, 10% polyethylene glycol (PEG) 300, 20% Tween 80.

### MTT Assay

The cytotoxicity of sorafenib in BV2 cells was assessed by evaluating mitochondrial arrest using the MTT (3-(4,5-dimethylthiazol-2-yl)-2,5-diphenyltetrazolium bromide) assay in 96-well plates. Cells (4 x 10^4^ cells/well) were treated for 6 or 24 h with sorafenib (0.1, 1, 5, 10, 20 or 25 μM) or vehicle (0.1, 1, 5, 10, 20 or 25 μM DMSO) without FBS. Then, MTT (0.5 mg/mL) was added and incubated for 3 h protected from light. Finally, the formazan crystals were dissolved with shaking in DMSO, and the absorbance at 570 nm was measured in a SPECTROstar Nano microplate reader (BMG Labtech, Germany).

### Cell Culture

The microglial cell line BV2 (a generous gift from Dr. Kyung-Ho Suk) was cultured in high-glucose DMEM (Invitrogen, Carlsbad, CA, USA) with 5% fetal bovine serum (FBS, Invitrogen) at 37°C and 5% CO_2_. Rat primary cortical astrocytes were isolated from postnatal day 1 Sprague Dawley rats as previously described (7, 18). In brief, the cortex was removed from the sacrificed mouse and dissociated into single cells in high-glucose DMEM supplemented with 10% FBS/penicillin-streptomycin solution. Cells plated in 75-T flasks were then incubated at 37°C with 5% CO_2_ for 2 weeks. Astrocytes were detached by agitating the 75-T flasks at 120 rpm for 2 h, and after removing the conditioned medium, the cells were centrifuged for 30 min at 2000 rpm and washed thrice with PBS. Finally, the cells were resuspended in high-glucose DMEM with 10% FBS/penicillin-streptomycin and aliquoted in 12-well plates.

### Reverse Transcription PCR (RT-PCR) and Real-Time PCR

TRIzol (Invitrogen) was used to extract total RNA from cells. For BV2 cells, Superscript cDNA Premix Kit II (GeNetBio, Daejeon, Korea) and Prime Taq Premix (GeNetBio) were used for RT-PCR. For primary astrocytes, Fast SYBR Green Master Mix (Thermo Fisher Scientific, CA, USA) and a QuantStudio 5 Real-Time PCR System (Thermo Fisher Scientific, San Jose, CA, USA) were used for real-time PCR. Normalization was performed according to the Gadph cycle threshold (Ct) value, and the fold change in sorafenib-treated cells was calculated relative to the vehicle-treated control. The sequences of the primers are given in **Tables 1** and **2**. The RT-PCR data for groups in which LPS treatment did not induce proinflammatory responses were excluded.

**Table 1:** Sequences of primers used for RT-PCR.

Gene name		Sequence
*IL-1β*	*Sense*	*5’-AGC TGG AGA GTG TGG ATC CC-3’*
	*Antisense*	*5’-CCT GTC TTG GCC GAG GAC TA-3’*
*IL-6*	*Sense*	*5’-CCA CTT CAC AAG TCG GAG GC-3’*
	*Antisense*	*5’-GGA GAG CAT TGG AAA TTG GGG T-3’*
*COX-2*	*Sense*	*5’-GCC AGC AAA GCC TAG AGC-3’*
	*Antisense*	*5’-GCC TTC TGC AGT CCA GGT TC-3’*
*iNOS*	*Sense*	*5’-CCG GCA AAC CCA AGG TCT AC-3’*
	*Antisense*	*5’-GCA TTT CGC TGT CTC CCC AA-3’*
*GAPDH*	*Sense*	*5’-CAG GAG CGA GAC CCC ACT AA-3’*
	*Antisense*	*5’-ATC ACG CCA CAG CTT TCC AG-3’*

**Table 2:** Sequences of primers used for real time-PCR.

Gene name		Sequence
*IL-1β*	*Sense*	*5’-TTG ACG GAC CCC AAA AGA TG-3’*
	*Antisense*	*5’-AGG ACA GCC CAG GTC AAA G -3’*
*IL-6*	*Sense*	*5’-CCA CGG CCT TCC CTA CTT C-3’*
	*Antisense*	*5’-TTG GGA GTG GTA TCC TCT GTG A-3’*
*COX-2*	*Sense*	*5’-CCA CTT CAA GGG AGT CTG GA -3’*
	*Antisense*	*5’-AGT CAT CTG CTA CGG GAG GA-3’*
*iNOS*	*Sense*	*5’-GGA TCT TCC CAG GCA ACC A-3’*
	*Antisense*	*5’-TCC ACA ACT CGC TCC AAG ATT-3’*
*GAPDH*	*Sense*	*5’-TGG GCT ACA CTG AGG ACC ACT-3’*
	*Antisense*	*5’-GGG AGT GTC TGT TGA AGT CG-3’*

### Immunocytochemistry (ICC)

Immunocytochemistry of BV2 cells was conducted according to a previously methodology (6, 7). In brief, cells were fixed for 10 min in 4% PFA, washed thrice with PBS, and then incubated with either anti-CD11b (Abcam, Cambridge, UK) and anti-p-STAT3^S727^ (Abcam) or anti-CD11b and anti-p-NF-κB^S536^ (Cell Signaling Technology, Danvers, MA, USA) antibodies overnight (**Table 3**). After washing the cells with PBS for 10 min, Alexa Fluor 488-conjugated anti-mouse and Alexa Fluor 555-conjugated anti-rabbit antibodies (1:200, Molecular Probes, USA) were incubated for 1 h at room temperature. After washing thrice with PBS for 10 min, the cells were mounted with DAPI (Vector Laboratories, CA, USA), and fluorescence microscopy images were acquired (DMi8, Leica Microsystems, Wetzlar, Germany) and analyzed using ImageJ.

**Table 3:** List of antibodies used in this study.

Primary antibodies							
Antigen	Host species		Dilution	Manufacturer	Catalog no.	Analysis
Iba-1	Rabbit polyclonal		1:500	Wako	019-19741	IF	
GFAP	Rabbit polyclonal		1:500	Neuromics	RA22101	IF	
IL-1β	Rabbit polyclonal		1:200	Abcam	AB9722	IF	
COX-2	Rabbit polyclonal		1:500	Abcam	AB15191	IF	
p-AKT^S473^	Rabbit polyclonal		1:500	Cell Signaling	9271	WB/IF	
AKT	Rabbit polyclonal		1:500	Cell Signaling	9272S	WB	
p-STAT3^S727^	Rabbit polyclonal		1:500	Abcam	AB86340	ICC/IF	
p-NF-κB^S536^	Rabbit polyclonal		1:500	Cell Signaling	3033S	ICC	
p-P38^T180/Y182^	Rabbit polyclonal		1:1000	Abcam	9211	WB	
P38	Rabbit polyclonal		1:1000	Abcam	9212	WB	
CD11b	Rat monoclonal		1:200	Abcam	AB8878	ICC	
**Secondary antibodies**							
**Antibody**			**Dilution**	**Manufacturer**	**Catalog no.**	** Analysis**	
Goat anti-rabbit IgG, Alexa Fluor 488			1:200	Invitrogen	A11008	IF	
Goat anti-rabbit IgG, Alexa Fluor 555			1:200	Invitrogen	A28180	IF, ICC	
Goat anti-chicken IgG, Alexa Fluor 488			1:500	Invitrogen	A11001	IF	
Goat anti-rat IgG, Alexa FITC			1:200	Invitrogen	A18866	ICC	
Goat anti-rabbit IgG, HRP conjugate			1:10000	Enzo	ADI-SAB-300-J	WB	
Goat anti-mouse IgG, HRP conjugate			1:10000	Enzo	ADI-SAB-100-J	WB	

### Wild-Type Mice

Adult wild-type C57BL6/J male mice (8 weeks old, 25-30 g; Orient-Bio Company, Gyeonggi-do, Korea) were housed in a pathogen-free facility with food and water *ad libitum* and a photoperiod of 12 h. In all experiments, mice were randomly allocated to the vehicle or sorafenib treatment group. To examine the preventive effects of sorafenib on LPS-induced neuroinflammatory responses, wild-type mice were intraperitoneally (i.p.) administered vehicle (5% DMSO, 10% PEG 300, 20% Tween 80) or sorafenib (10 mg/kg) daily for 3 consecutive days. Thirty minutes after the last injection, LPS (10 mg/kg, i.p.) or PBS was administered, and 8 h later, the mice were anesthetized and transcardially perfused with PBS followed by 4% paraformaldehyde (PFA). To assess the therapeutic effects of sorafenib on LPS-evoked neuroinflammatory responses, wild-type mice were administered LPS (10 mg/kg, i.p.) or PBS, and 30 min later, sorafenib (10 mg/kg, i.p.) or vehicle was administered three times at 2-h intervals (i.e., sorafenib was injected 30 min, 2.5 h, and 4.5 h after LPS or PBS injection). Eight hours after LPS injection, the mice were anesthetized and transcardially perfused. The *in vivo* experimental design is summarized in **Figures 3A**, **6A**, **8A**.

### 5xFAD Mice

5xFAD mice were used to determine the effects of sorafenib on Aβ-induced neuroinflammatory responses; these mice carry five familial AD mutations (APPSw, Lon, Flo, PS1M146L, L286V) under the control of the Thy1 promoter, resulting in overexpression of Aβ. 5xFAD mice (Stock No. 34848-JAX; B6.Cg-Tg (APPSwFlLon,PSEN1*M146L*L286V)6799Vas/Mmjax) were purchased from Jackson Laboratory (Bar Harbor, ME, USA). Genotyping of each mouse was performed using genomic DNA extracted from a tail snip. Only male mice were used in this study.

### Immunofluorescence Staining (IF)

The brains of wild-type and 5xFAD mice fixed as described above were sectioned at a thickness of 30 μm with a cryostat microtome. The sections were incubated with 10% normal goat serum (Vector Laboratories) for 1 h at room temperature, immunostained overnight at 4°C with primary antibodies (Iba-1, GFAP, COX-2, p-AKT, p-STAT3), and incubated with secondary antibodies for 2 h at room temperature. Images of sections mounted on glass slides with DAPI (Vector Laboratories) were acquired by fluorescence microscopy (DMi8, Leica Microsystems, Wetzlar, Germany) and analyzed by ImageJ (NIH). Quantification was performed using 2-3 brain slices per mouse and a total of 18-24 brain images/per group (4 mice/group). The primary and secondary antibodies are listed in **Table 3**.

### Western Blotting (WB)

The potential effects of sorafenib on LPS-mediated AKT and P38 signaling were assessed in BV2 microglial cells treated with 200 ng/ml LPS or PBS for 45 min followed by 5 μM sorafenib or vehicle (1% DMSO) for 5.5 hr. The cells were then incubated in lysis buffer (ProPrep, iNtRON Biotechnology, Inc., Seongnam, Korea) supplemented with protease and phosphatase inhibitor for 5 min, followed by centrifugation at 12,000 rpm. The supernatants were collected, and protein concentrations were measured by the BSA protein assay. The quantified proteins were mixed with 4x SDS sample buffer and separated by electrophoresis on an 8% SDS-PAGE gel for 2 h. The proteins were then transferred to a polyvinylidene difluoride (PVDF) membrane, followed by blocking with 5% skim milk (for total AKT and total P38) or 5% BSA (for p-AKT and p-P38) for 1 h. Next, the membranes were incubated with primary antibodies at 4°C overnight, washed for 5 min four times with TBST, and incubated with peroxidase-conjugated secondary antibodies for 1 h at RT. Finally, the membrane was washed with TBST for 5 min, and the target proteins were visualized using ECL Western Blotting Detection Reagent (GE Healthcare, Chicago, IL, USA). Fusion Capt Advance software (Vilber Lourmat) was used for image analysis. The primary and secondary antibodies are listed in **Table 3**.

### Statistical Analysis

Comparisons of two groups were performed with unpaired two-tailed t-tests with Welch’s correction; multiple comparisons were performed by one-way ANOVA (parametric or non-paramatric) (Prism 7, GraphPad Software, USA). Post hoc analysis was conducted with Tukey’s or Dunn’s multiple comparison test; p < 0.05 was considered significant. The normal distribution of data from *in vitro* and *in vivo* experiments was verified using the Kolmogorov-Smirnov or Shapiro-Wilk normality test (**Supplementary Tables 2, 3**). Data are presented as the mean ± SD (*p < 0.05, **p < 0.01).

## Results

### Sorafenib Decreases LPS-Induced COX-2 and IL-1β mRNA Levels in Microglial Cells

The anti-cancer drug sorafenib is a multi-target kinase inhibitor (**Figure 1A**). In the present study, we examined the effects of sorafenib on neuroinflammation. First, mitochondrial arrest resulting from sorafenib cytotoxicity was assessed *in vitro* in BV2 microglial cells using the MTT assay. No cytotoxicity was observed after 6 or 24 h at sorafenib concentrations of 0.1 to 10 μM, but cytotoxicity was evident at 20–25 μM sorafenib (**Figures 1B, C**). Based on these data, we selected a sorafenib concentration of 5 μM as an intermediate concentration with no apparent cytotoxicity for all subsequent *in vitro* experiments.

To investigate the effects of sorafenib on the proinflammatory response induced by LPS, BV2 microglial cells were exposed first to vehicle (1% DMSO) or 5 μM sorafenib for 30 min and then 200 ng/ml LPS or PBS for 5.5 h. Proinflammatory cytokine levels were assessed by RT-PCR (**Figure 1D**). Sorafenib pretreatment prevented the increase in COX-2 and IL-1β mRNA levels evoked by LPS but did not alter IL-6 and iNOS mRNA levels (**Figures 1E, F** and **Supplementary Figure 1A**).

To examine the potential therapeutic influence of sorafenib on the proinflammatory response, BV2 microglial cells were exposed first to 200 ng/ml LPS or PBS for 30 min and then to vehicle (1% DMSO) or 5 μM sorafenib for 5.5 h (**Figure 1G**). Sorafenib posttreatment significantly reduced the increase in COX-2 and IL-1β mRNA levels under LPS stimulation but did not alter IL-6 and iNOS levels (**Figures 1H, I** and **Supplementary Figure 1B**). Thus, either pre-treatment or post-treatment with sorafenib can regulate the LPS-evoked increase in proinflammatory cytokines in microglial cells.

### Sorafenib Reduces LPS-Induced COX-2 mRNA Levels in Primary Astrocytes

The effects of sorafenib on the proinflammatory response were further investigated in primary astrocytes. The cells were first treated with 200 ng/ml LPS or PBS for 30 min and then vehicle (1% DMSO) or 5 μM sorafenib for 5.5 h. Real-time PCR was performed to assess proinflammatory cytokine levels. Sorafenib posttreatment significantly decreased the LPS-induced increase in COX-2 mRNA expression but not IL-1β, IL-6 and iNOS mRNA levels (**Figure 1J**). In summary, sorafenib appears to selectively regulate the LPS-induced increase in COX-2 mRNA expression in primary astrocytes.

### Sorafenib Suppresses LPS-Induced AKT/P38 Phosphorylation and Nuclear STAT3/NF-kB Phosphorylation

**Figure 2:**
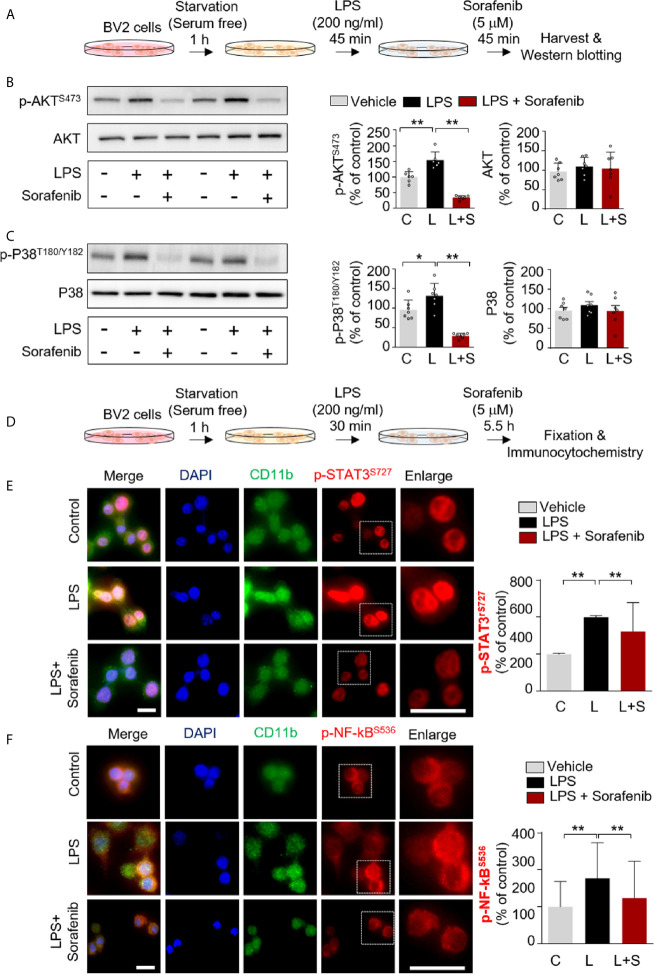
Sorafenib downregulates the LPS-induced increases in p-AKT/p-P38 and nuclear p-STAT3/p-NF-kB levels. **(A)** Scheme for sequential treatment of BV2 cells with LPS and sorafenib. **(B, C)** Western blotting analysis of BV2 cells treated as described in **(A)** with anti-p-AKT^S473^, anti-AKT, anti-p-P38^T180/Y182^, and anti-P38 antibodies (n=6/group). **(D)** Scheme for sequential treatment of BV2 cells with LPS and sorafenib. **(E, F)** Immunocytochemistry analysis of BV2 cells treated as described in **(D)** with anti-CD11b and anti-p-STAT3^S727^ antibodies (number of cells (n); Vehicle, n=428; LPS, n=357; LPS+sorafenib, n=361) or anti-CD11b and anti-p-NF-kB^Ser536^ antibodies (Vehicle, n=461; LPS, n=584; LPS+sorafenib, n=416). *p < 0.05, **p < 0.01, Scale bar = 20 μM.

AKT and the MAPK signaling kinase P38 play important roles in glial cell activation by modulating the secretion of proinflammatory cytokines (19). We recently reported that in BV2 microglial cells, another anti-cancer drug and multikinase inhibitor, regorafenib, alters AKT and P38 signaling and LPS-induced neuroinflammation (8). To assess the ability of sorafenib to regulate AKT and/or P38 signaling *in vitro*, BV2 microglial cells were treated with 200 ng/ml LPS or PBS for 45 min before treatment with 5 μM sorafenib or vehicle (1% DMSO) for 45 min. AKT and P38 phosphorylation were measured by Western blotting of total extracted proteins with anti-p-AKT^S473^/AKT and anti-p-P38^T180/Y182^/P38 antibodies (**Figure 2A**). Sorafenib posttreatment significantly decreased the LPS-induced increases in p-AKT^S473^ and p-P38^T180/Y182^ without changing the total levels of AKT and P38 induced by LPS in this cell line (**Figures 2B, C** and **Supplementary Figure 2**).

We then investigated the potential involvement of the transcription factors STAT3 and NF-kB in sorafenib-associated neuroinflammatory responses. BV2 microglial cells were first treated with 200 ng/ml LPS or PBS for 30 min before treatment with 5 μM sorafenib or vehicle (1% DMSO) for 5.5 h (**Figure 2D**). Subsequent immunocytochemistry analysis with anti-CD11b and anti-p-STAT3^S727^ or anti-p-NF-κB^S536^ antibodies revealed that sorafenib posttreatment significantly reduced the LPS-induced increase in nuclear p-STAT3^S727^ (**Figure 2E**). Interestingly, sorafenib posttreatment also significantly decreased LPS-induced nuclear p-NF-kB^S536^ levels (**Figure 2F**). In summary, sorafenib affects LPS-induced p-AKT/p-P38-linked signaling and its associated transcription factors, p-STAT3 and p-NF-kB, to modulate neuroinflammatory responses in microglial cells.

### Sorafenib Pretreatment Inhibits LPS-Induced Microgliosis in Wild-Type Mice

**Figure 3:**
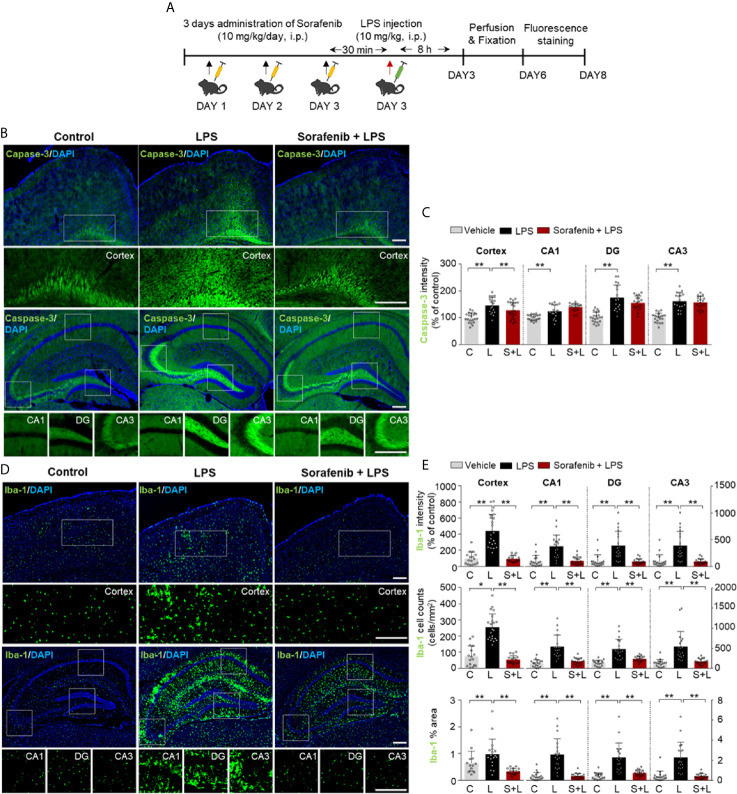
Pretreatment of sorafenib inhibits LPS-induced microgliosis in wild-type mice. **(A)** Scheme for treatment of wild-type mice with sorafenib followed by LPS. **(B, D)** Immunofluorescence staining of brain slices from wild-type mice treated as described in **(A)** with an anti-caspase-3 and anti-Iba-1 antibody. **(C, E)** Quantification of the data in **(B, D)** (analyzed number of brain slices/images (n); Caspase-3: Vehicle, n=21; LPS, n=18; Sorafenib + LPS, n=21, Iba-1: Vehicle, n=18; LPS, n=19; Sorafenib + LPS, n=19). *p < 0.05, **p < 0.01. Scale bar = 200 μM.

To examine the effects of sorafenib on LPS-evoked gliosis *in vivo*, we selected a sorafenib dose of 10 mg/kg based on previous studies (20–22). To assess the potential toxicity of this dose of sorafenib *in vivo*, wild-type mice were intraperitoneally (i.p.) injected with sorafenib (10 mg/kg/day) or vehicle daily for 3 consecutive days. Thirty minutes after the final injection of sorafenib, LPS (10 mg/kg, i.p.) or PBS was administered, and immunofluorescence staining of brain sections was performed with an antibody against caspase-3, a marker of apoptotic cell death (**Figure 3A**). We found that sorafenib pretreatment significantly decreased the LPS-induced increase in caspase-3 levels in the cortex but had no significant effect in the hippocampus (**Figures 3B, C**). According to these results, 10 mg/kg sorafenib does not appear to have cytotoxic effects in the brain.

Next, to determine the effects of sorafenib on LPS-induced microglial activation *in vivo*, we assessed levels of ionized calcium-binding adapter molecule 1 (Iba-1), a critical marker of microglial activation *in vivo* that initiates neuroinflammation defense mechanisms (23). Pretreatment of sorafenib significantly decreased the LPS-induced increase in Iba-1 immunofluorescence intensity in the cortex and hippocampus (CA1, DG, and CA3) (**Figures 3D, E**). Consistent with this finding, the number of Iba-1-positive cells and the percentage of the area that was stained in the cortex and hippocampus were significantly reduced in mice administered sorafenib after LPS induction (**Figures 3D, E**). Thus, sorafenib downregulates LPS-induced increases in microglial kinetics, morphological activity, and migration to sites of inflammation in the brain.

### Pretreatment of Sorafenib Suppresses LPS-Induced Astroglial Kinetics, Morphological Activity, and Migration and COX-2 Levels in Wild-Type Mice

**Figure 4:**
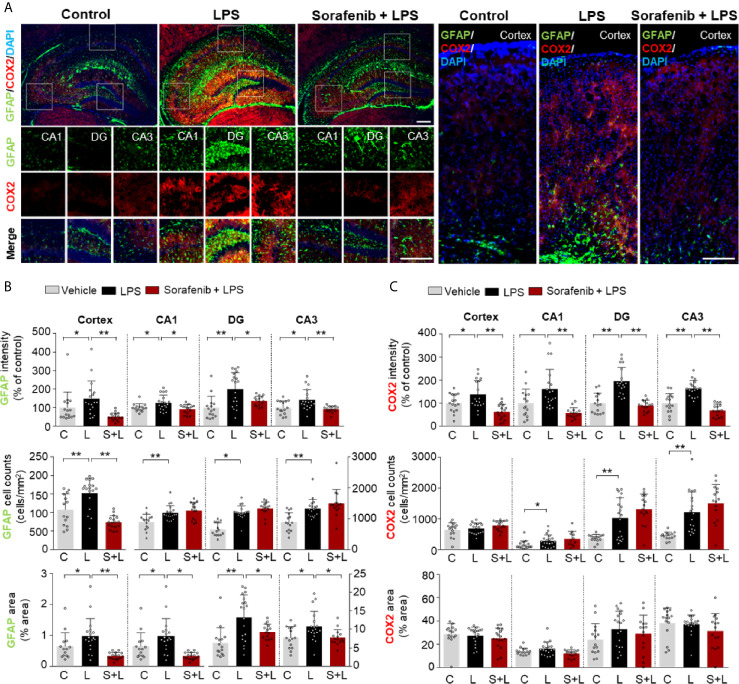
Pretreatment of sorafenib suppresses LPS-induced astrogliosis and COX-2 levels in wild-type mice. **(A)** Immunofluorescence staining with anti-GFAP and anti-COX-2 antibodies of brain slices from wild-type mice treated with sorafenib followed by LPS. **(B, C)** Quantification of the data in **(A)** (analyzed number of brain slices/images (n); GFAP: Vehicle; n=16; LPS, n=18; Sorafenib + LPS, n=17, COX-2: Vehicle, n=16; LPS, n=18; Sorafenib + LPS, n=17). *p < 0.05, **p < 0.01, Scale bar = 200 μM.

Astrocytes are neuron-supporting cells that modulate the LPS-mediated neuroinflammatory response by regulating nervous system repair (24). Astrocyte activation in response to infection or inflammation cues is essential for pathogen clearance and proinflammatory cytokine release and involves changes in astrocyte kinetics, morphology, and migration (25). To determine if sorafenib alters LPS-evoked astrogliosis *in vivo*, glial fibrillary acidic protein (GFAP) immunofluorescence intensity, the number of GFAP-activated cells, and the percentage of GFAP-stained area were measured in brain sections from wild-type mice injected as described above with 10 mg/kg sorafenib or vehicle followed by 10 mg/kg LPS or PBS. Pretreatment of sorafenib significantly reduced the LPS-induced increases in GFAP immunofluorescence intensity and area of staining in the cortex and hippocampus (CA1, DG, and CA3) (**Figures 4A, B**). However, the LPS-induced increase in the number of GFAP-labeled cells was only decreased in the cortex by sorafenib treatment (**Figures 4A, B**). These data indicate that sorafenib reduces LPS-induced activation of astrocyte kinetics, morphological changes, and atrocytic migration in the wild-type mouse brain.

To verify the effects of sorafenib on the LPS-induced increase in COX-2 levels observed in **Figure 1**, brain sections from wild-type mice treated as described in **Figure 3A** were subjected to immunofluorescence staining with an anti-COX-2 antibody. Pretreatment of sorafenib significantly reduced the LPS-induced increase in COX-2 immunofluorescence intensity in the cortex and hippocampus (CA1, DG, and CA3) (**Figures 4A–C**). These data confirm that sorafenib regulates LPS-mediated COX-2 levels in wild-type mice.

### Pretreatment of Sorafenib Decreases AKT and STAT3 Phosphorylation in Wild-Type Mice

**Figure 5:**
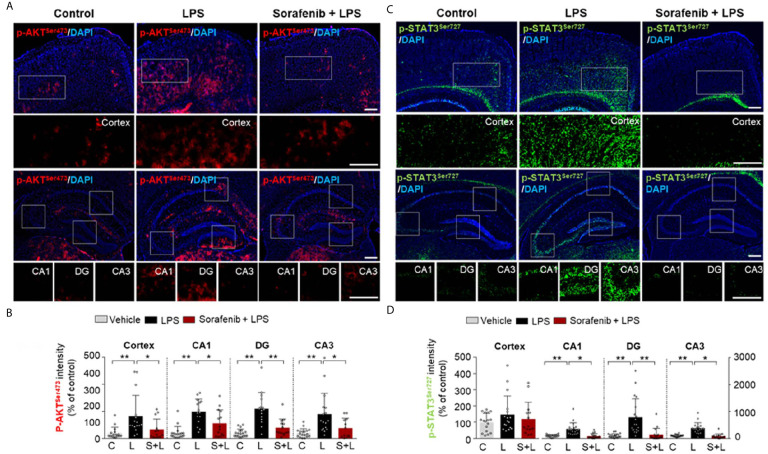
Pretreatment of sorafenib decreases LPS-mediated AKT and STAT3 phosphorylation in wild-type mice. **(A, C)** Immunofluorescence staining with anti-p-AKT^S473^ and anti-p- STAT3^S727^ antibody of brain slices from wild-type mice treated with sorafenib followed by LPS. **(B, D)** Quantification of the data in **(A, C)** (analyzed number of brain slices/images (n); p-AKT ^S473^: Vehicle, n =16; LPS, n=16; Sorafenib + LPS, n=14, p-STAT3 ^S727^, Vehicle, n=23; LPS, n=22; Sorafenib + LPS, n=20). *p < 0.05, **p < 0.01, Scale bar = 200 μM.

The induction of AKT and STAT3 signaling by LPS has been linked to the regulation of microglial and astrocyte activation *in vivo* (26). The effects of sorafenib on neuroinflammation-mediated phosphorylation of AKT and STAT3 were assessed in wild-type mice treated as described in **Figure 3A** with 10 mg/kg sorafenib or vehicle followed by 10 mg/kg LPS or PBS. Immunofluorescence staining of brain sections was performed using anti-p-AKT^S473^ and anti-p-STAT3^S727^ antibodies. Pretreatment of sorafenib significantly reduced p-AKT^S473^ levels in the cortex and hippocampus stimulated by LPS (**Figures 5A, B**). In addition, pretreatment of sorafenib had no effect on LPS-induced p-STAT^S727^ levels in the cortex but significantly reduced hippocampal LPS-induced p-STAT^S727^ levels compared with LPS treatment (**Figures 5C, D**). Thus, sorafenib pretreatment modulates the phosphorylation of AKT and STAT3 in a brain region-specific manner in wild-type mice injected with LPS.

### Posttreatment of Sorafenib Inhibits LPS-Induced Microgliosis and Astrogliosis in Wild-Type Mice

**Figure 6:**
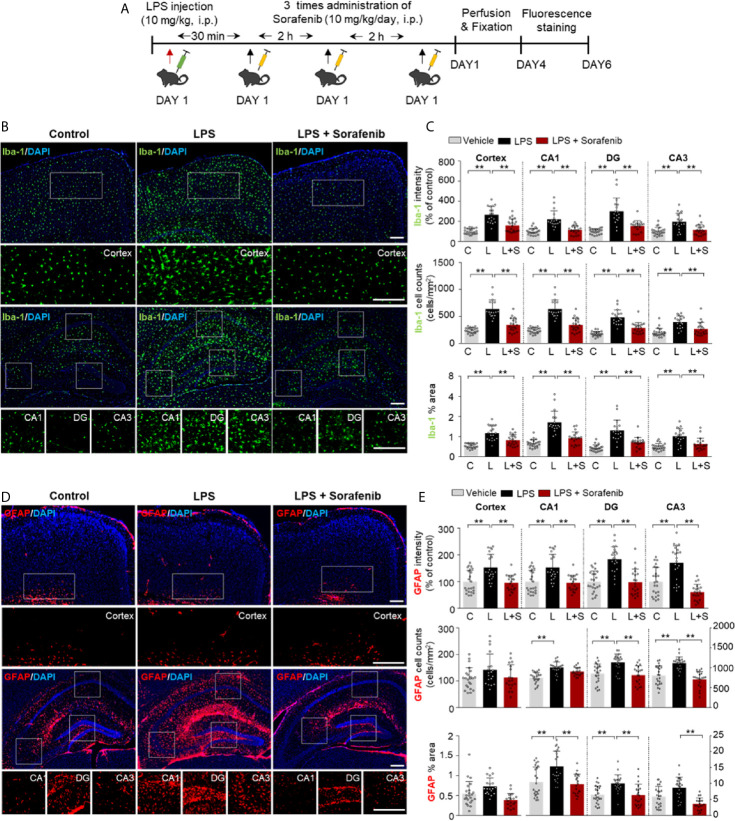
Posttreatment of sorafenib inhibits LPS-induced microgliosis and astrogliosis in wild-type mice. **(A)** Scheme for the treatment of wild-type mice with LPS followed by sorafenib. **(B, D)** Immunofluorescence staining with anti-Iba-1 and anti-GFAP antibodies of brain slices from wild-type mice treated as described in **(A)**. **(C, E)** Quantification of the data in **(B, D)** (analyzed number of brain slices/images (n); Iba-1: Vehicle, n=21; LPS, n=23; LPS + Sorafenib, n=18, GFAP, Vehicle, n=23; LPS, n=22; LPS + Sorafenib, n=20). **p < 0.01. Scale bar = 200 μM.

Since exposure to sorafenib before LPS injection regulates LPS-mediated gliosis *in vivo*, we investigated whether posttreatment of sorafenib alters LPS-evoked neuroinflammation. As described in **Figure 6A**, wild-type mice were i.p. injected with 10 mg/kg LPS or PBS followed 30 min later by three i.p. injections of 10 mg/kg sorafenib or vehicle at 2 h intervals. Eight hours after the initial LPS or PBS injection, the mice were sacrificed, and immunofluorescence staining of brain sections was performed with an anti-Iba-1 or anti-GFAP antibody (**Figure 6A**). Posttreatment of sorafenib significantly decreased the LPS-induced increase in Iba-1 immunofluorescence intensity in the cortex and hippocampus (CA1, DG, and CA3) (**Figures 6B, C**). The number of Iba-1-positive cells and the percentage of the stained area in the cortex and hippocampus were also significantly decreased when LPS injection was followed by sorafenib treatment in wild-type mice (**Figures 6B, C**). Similar to Iba-1, posttreatment of sorafenib significantly reduced the LPS-induced increase in GFAP immunofluorescence intensity in the cortex and hippocampus (**Figures 6D, E**). In addition, the LPS-mediated increase in GFAP-labeled cells and the percent area of staining were significantly decreased in the hippocampus but not the cortex (**Figures 6D, E**) in the presence of sorafenib. These data indicate that posttreatment with sorafenib downregulates the neuroinflammatory response by inhibiting glial activation in the brain.

### Posttreatment of Sorafenib Regulates LPS-Induced AKT Phosphorylation in Wild-Type Mice

**Figure 7:**
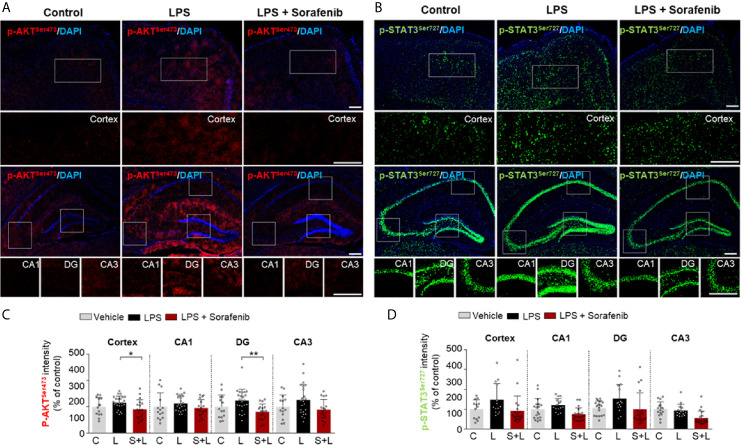
Posttreatment of sorafenib diminishes LPS-evoked AKT phosphorylation in wild-type mice. **(A**, **C)** Immunofluorescence staining with anti-p-AKT^S473^ and anti-p-STAT3^S727^ antibodies of brain slices from wild-type mice treated with LPS followed by sorafenib. **(B, D)** Quantification of the data in **A, C** (analyzed number of brain slices/images (n); p-AKT ^S473^: Vehicle, = 14; LPS, n=24; LPS + Sorafenib, n=18, p-STAT3 ^S727^, Vehicle, n=13; LPS, n=14; LPS + Sorafenib, n=20). *p < 0.05, **p < 0.01, Scale bar = 200 μM.

To determine if posttreatment of sorafenib affects LPS-evoked neuroinflammatory-associated signaling, wild-type mice were injected with 10 mg/kg LPS or PBS and then injected with 10 mg/kg sorafenib or vehicle as described in **Figure 6A**. Immunofluorescence staining of brain sections from the mice was performed with anti-p-AKT^S473^ and anti-p-STAT3^S727^ antibodies. Posttreatment with sorafenib significantly decreased LPS-induced increase in AKT phosphorylation in cortex and DG but not CA1 and CA3 (**Figures 7A, B**). Moreover, posttreatment of sorafenib had no effect on LPS-induced p-STAT^S727^ levels in the cortex and hippocampus (**Figures 7C, D**). These data indicate that posttreatment with sorafenib modulates AKT signaling but not STAT3 to alter LPS-mediated neuroinflammation in wild-type mice.

### Sorafenib Upregulated the LPS-Mediated Decrease in Shank-1 Intensity in Wild-Type Mice

Systemic inflammation and neuroinflammation can impact cognitive and synaptic function (27). Since pre-and posttreatment of sorafenib decreased LPS-mediated gliosis in wild-type mice, we examined whether sorafenib modulates learning and memory-related proteins. For these experiments, wild-type mice were injected with sorafenib followed by LPS as described in **Figure 3A**, and brain sections were immunostained with anti-synaptophysin (a presynaptic marker), anti-PSD-95 (a postsynaptic marker), or anti-shank-1 antibodies. Sorafenib pretreatment did not alter the immunofluorescence intensity of synaptophysin in wild-type mice treated with LPS (**Supplementary Figures 3A, B**), and a trend toward increased PSD-95 immunofluorescence intensity was observed (**Supplementary Figures 3C, D**). Interestingly, pre-exposure to sorafenib rescued the LPS-induced decrease in shank-1 immunofluorescence intensity in the cortex and hippocampal DG (**Supplementary Figures 3E, F**). These data suggest that sorafenib pretreatment may positively or negatively modulates synaptic function in LPS-induced wild-type mice.

### Sorafenib Suppresses Aβ-Mediated Astrogliosis in 5xFAD Mice

**Figure 8:**
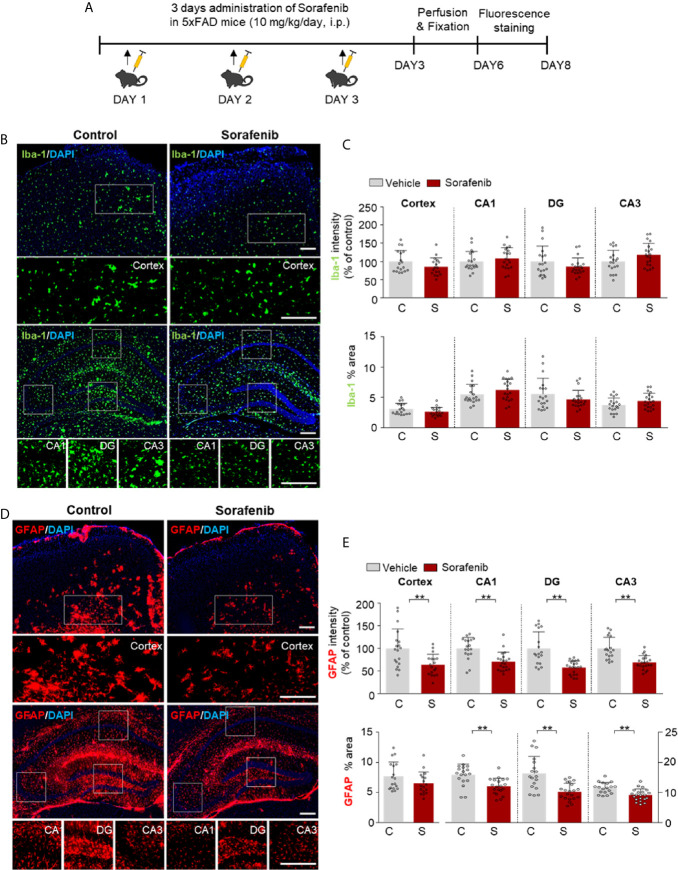
Sorafenib suppresses Aβ-mediated astrogliosis in 5xFAD mice. **(A)** Scheme for the treatment of 5xFAD mice with sorafenib. **(B, D)** Immunofluorescence staining with anti-Iba-1 and anti-GFAP antibodies of brain slices from 5xFAD mice treated as described in **(A)**. **(C, E)** Quantification of the data in **(B, D)** (analyzed number of brain slices/images (n); Vehicle: =21, Sorafenib: n= 18). **p < 0.01. Scale bar = 200 μM.

Both pre- and post-treatment with sorafenib effectively downregulated LPS-mediated neuroinflammation *in vitro* and *in vivo*. To determine the effects of sorafenib on neuroinflammatory responses in a mouse model of Alzheimer’s disease (AD), 5xFAD mice were treated with sorafenib (10 mg/kg/day, i.p.) or vehicle daily for 3 consecutive days, and immunofluorescence staining were conducted with an anti-Iba-1 or anti-GFAP antibody (**Figure 8A**). Three consecutive days of sorafenib administration did not alter Iba-1 immunofluorescence intensity and the percent area stained (**Figures 8B, C**). However, Aβ-induced GFAP immunofluorescence intensity in the cortex and hippocampus was significantly reduced (CA1, DG, and CA3) (**Figures 8D, E**). Consistent with these observations, sorafenib administration significantly decreased the percent area of GFAP in the hippocampus (CA1, DG, and CA3) but not the cortex (**Figures 8D, E**). These data suggest that 3 consecutive days of sorafenib administration selectively modulates Aβ-mediated astrogliosis in the brains of 5xFAD mice.

## Discussion

In this study, we demonstrated that sorafenib, a multikinase inhibitor and anti-cancer drug, decreases the levels of proinflammatory cytokines as well as microglial and astrocyte activation induced by LPS. In BV2 microglial cells, sorafenib diminished the LPS-induced increases in COX-2 and IL-1β levels by modulating P38/AKT and NF-kB/STAT3 signaling. Sorafenib also decreased the LPS-induced increase in COX-2 levels in primary astrocytes. *In vivo*, pre- and posttreatment of sorafenib decreased LPS-induced changes in microglial kinetics, number, and morphology. LPS-induced astrocyte activation was also reduced by pre- or posttreatment of sorafenib, although pretreatment of sorafenib effect on astrocyte number. In wild-type mice, sorafenib administration significantly reduced the LPS-induced increase in COX-2 levels by altering AKT/STAT3 signaling. Moreover, in 5xFAD mice, sorafenib treatment (daily for 3 days) significantly suppressed astrogliosis but not microgliosis. Taken together, our findings suggest that sorafenib could be a novel therapy for relieving neuroinflammatory response-associated glial activation in the brain.

We and others have recently found that several small compounds and herbal extracts can modulate LPS-evoked neuroinflammatory responses *in vitro* and *in vivo*. For instance, the multitarget kinase inhibitor dasatinib, whose targets include Bcr-Abl and the Src kinase family, decreases LPS-induced COX-2 and IL-6 levels *via* the AKT/STAT3 signaling pathway (6). Apamin (APM), a selective antagonist of small conductance calcium-activated potassium (SK) channels, inhibits LPS-stimulated TLR4 activation throughout CaMKII/ERK and NF-kB/STAT3 phosphorylation in BV2 and primary microglial cells (28). In addition, ALWPs, a mixture of Antler and LWPs, suppresses the LPS-induced increase in IL-1β by downregulating FAK/NF-kB signaling pathways (29). These findings suggest that LPS-mediated neuroinflammation could be regulated by various signaling pathways linked to drug targets.

In the present study, we investigated the effects of sorafenib on neuroinflammation. The kinase targets of sorafenib include Raf-1, VEGFR2 (Flk1), and PDGFRβ (30), which are all potent inducers of proinflammatory cytokine release from microglial cells and play significant roles in microglial-associated neuroinflammatory responses (31–33). For instance, high-dose infusion of VEGF-A, which binds VEGFR2, leads to the release of the proinflammatory cytokine MIP-1 alpha in the adult rat cortex (34). In cultured T cells, VEGF treatment induces the secretion of interferon gamma (IFN-γ) (35). In addition, oral administration of didymin (an activator of Raf-1 kinase inhibitor protein (RKIP)) increases levels of TNF-α, IL-6, and IL-1β in liver tissues and RAW 264.7 cells (36). In the brains of mice, intra-ipsilateral and contralateral infusion of the PDGFRβ inhibitor Greevec decreases the intracerebral hemorrhage-induced increase in TNF-α levels (37). Overall, these findings suggest that the effects of sorafenib on LPS-induced proinflammatory cytokine release may occur *via* modulation of Raf-1, VEGFR and/or PDGFRβ signaling.

The effects of multikinase inhibitors, including those targeting VEGFRs and PDGFRβ (e.g., axitinib, nintedanib, dabrafenib, regorafenib), are known to be associated with proinflammatory cytokine release (38). For example, treatment of primary motor cortical neurons with dabrafenib (an inhibitor of C-Raf/Raf-1 and B-Raf) reduces TNF-α and IL-12 levels (39). Regorafenib, another multikinase inhibitor that inhibits VEGFR2 and PDGFRβ, strongly reduces the LPS-induced increases in COX-2, IL-1β, IL-6, and TNF-α mRNA expression in BV2 microglial cells (8). IL-6, TNF-α, and IFN-γ expression in melanoma cells are also suppressed by axitinib, a selective inhibitor of VEGFRs and PDGFRs (40). In addition, the VEGFR, PDGFR and fibroblast growth factor receptor (FGFR) inhibitor nintedanib reduces the secretion of several proinflammatory and fibrotic cytokines (IL-1β, IL-8, IL-10 and CXCL13) in M1 macrophages (41). Here, we found that pre- and post-treatment with sorafenib significantly reduced the increases in COX-2 and IL-1β mRNA levels in BV2 microglial cells treated with LPS (**Figure 1**). Taken together, the present findings and previous work suggest that sorafenib downregulates proinflammatory cytokine mRNA levels by inhibiting VEGFR2 and/or PDGFRβ activation. In future work, we will determine whether sorafenib regulates LPS-induced proinflammatory cytokine release by inhibiting Raf-1, VEGFR2, and PDGFRβ simultaneously or individually.

Astrocytes control multiple processes in the CNS, including synaptogenesis, neuronal differentiation, neuronal survival and neuroinflammation (42). Under conditions of inflammation, astrocytes communicate with microglia and regulate extracellular proinflammatory cytokine homeostasis (42). Previous studies have reported that proinflammatory cytokine levels are elevated in LPS-induced rat and primary astrocytes (6, 7). Interestingly, the mRNA and protein levels of VEGFRs are elevated in glioblastoma cells, and soluble VEGFR1 is increased in astrocytic tumor cells compared with normal astrocytes (43, 44). Similarly, phosphorylation of PDGFRβ (tyrosine 75) is significantly increased in astrocytes located near breast cancer cells compared with normal astrocytes (45). Moreover, we recently demonstrated that regorafenib, an inhibitor of VEGFRs and PDGFRβ, suppresses the LPS-induced increase in COX-2 mRNA expression in primary astrocyte culture (8). However, the functions of VEGFR2 and/or PDGFRβ remain to be verified using target-specific blockade or epigenetic knockdown in astrocytes. In the current study, sorafenib only decreased the LPS-induced increase in mRNA levels of COX-2 mRNA and not other proinflammatory cytokines in primary astrocytes. Why do the multikinase inhibitors sorafenib and regorafenib affect only LPS-induced COX-2 mRNA levels? It is possible that multikinase inhibitors mainly inhibit the activation of VEGFRs and PDGFRβ in astrocytes and critically regulate COX-2 gene expression in response to LPS. Thus, sorafenib may modulate LPS-induced glial proinflammatory cytokine expression by regulating the activities of VEGFRs and/or PDGFRβ.

LPS stimulates Toll-like receptor 4 (TLR4) signaling pathways to induce proinflammatory cytokine release by microglia and astrocytes (5). AKT and MAPK signal transduction are among the main signaling pathways activated by TLR4 and share signals with VEGFR2 and/or PDGFRβ stimulation (5, 46, 47). AKT is a serine/threonine-specific protein kinase that is phosphorylated at S473 in the C-terminus or T308 in the kinase domain and plays a key role as a multiple activator of LPS-induced signaling in microglia (48). Vorolanib, an inhibitor of VEGFRs and PDGFRs, significantly reduces p-VEGFR2 and p-AKT levels in a dose-dependent manner in human umbilical vein endothelial cells (HUVECs) (49). Interestingly, combination treatment with vorolanib and gefitinib (an EGFR inhibitor) increases EGFR mutation and inhibits angiogenesis by downregulating VEGFR-linked AKT-STAT3 signaling (49). In addition, the VEGFRs and PDGFRβ inhibitor regorafenib significantly reduces the increase in AKT phosphorylation in LPS-stimulated BV2 cells (8), and the resulting decrease in AKT activation in turn decreases the induction of IL-1β and COX-2 mRNA expression (50). Here, we showed that sorafenib, an inhibitor of VEGFRs and PDGFRβ, also significantly reduces AKT phosphorylation in BV2 cells (**Figure 2B**), suggesting that sorafenib modulates LPS-mediated AKT signaling to alter neuroinflammatory responses in microglia.

After identifying the involvement of LPS-linked AKT signaling in the effects of sorafenib in microglia, we next investigated whether sorafenib modulates P38 signaling, a key pathway in the production of inflammatory mediators (51). For instance, activation of p-P38^T180/Y182^ by Ras-Raf kinases stimulates the release of proinflammatory cytokines from microglia (52, 53), and the VEGFRs inhibitor nintedanib decreases p-P38 immunoreactivity in GC7901 and MKN45 cells (54). Co-treatment of non-small cell lung carcinoma (NSCLC) cells with VEGF and vandetanib or axitinib, both VEGFR inhibitors, significantly suppresses the phosphorylation of P38 in a dose-dependent manner (55). In addition, the PDGFRβ inhibitor AG1295 decreases P38 phosphorylation in aortic vascular smooth muscle cells (56). Interestingly, in the present study, sorafenib suppressed the LPS-induced increase in p-P38^T180/Y182^ in BV2 cells (**Figure 2C**). These data indicate that sorafenib regulates P38 phosphorylation by inhibiting VEGFRs and/or PDGFRβ-linked P38 signaling in microglial cells. Further studies will reveal whether sorafenib affects other signaling pathways linked to VEGFRs and/or PDGFRβ in response to neuroinflammatory responses in microglia.

The expression of proinflammatory cytokines is transcriptionally regulated by phosphorylated STAT3 and NF-kB in the nucleus in microglia (57). Several studies have demonstrated that activated P38 and AKT phosphorylate STAT3 at S727 and/or NF-kB at S536 (6, 7). Phosphorylation at S727 enhances the transcriptional activity of STAT3 as well as the transcript levels of several proinflammatory cytokines (i.e., TNF-α, IL-1β, IL-6, and COX-2) in microglial cells (6, 7). Similarly, phosphorylation of p65, a component of NF-kB, at S536 induces NF-kB import into the nucleus and activation of LPS-mediated proinflammatory cytokine transcription (58). A PDGFβ-specific inhibitor, TKI258, reduces the PDGF-β-induced increase in STAT3 phosphorylation in MiaPaCa2 pancreatic cancer cells and endothelial cells (59). Interestingly, VEGFR2 overexpression increases the DNA binding affinity of NF-kB, whereas the VEGFR2 inhibitors sunitinib and bevacizumab suppress DNA binding by NF-kB in endothelial cells (60). Moreover, the VEGFRs and PDGFRβ inhibitor vorolanib diminishes STAT3 and NF-kB phosphorylation in a dose-dependent manner in NSCLC cells and xenograft mice (49). We recently demonstrated that the multikinase inhibitor regorafenib decreases nuclear p-STAT3^S727^ and p-NF-kB^S536^ levels in LPS-treated BV2 cells (8). Consistent with these observations, in the present study, sorafenib significantly decreased the LPS-stimulated increases in p-STAT3^S727^ and p-NF-kB^S536^ levels in BV2 cells. Thus, it is possible that sorafenib suppresses LPS-evoked p-STAT3 and p-NF-kB levels by inhibiting VEGFRs and PDGFRβ signaling in microglia.

*In vivo*, neuroinflammation is initiated by the activation of microglia and astrocytes to protect damaged neurons (2). Iba-1 is specifically expressed in microglia and is upregulated during microglial activation (i.e., changes in microglial morphology and location) in the brain (2). Similar to microglia, astrocytes can change their morphology, size, and mobility and become hypertrophic and hyperplastic upon LPS injection or other injury (61). In assessing the effects of sorafenib pre- or posttreatment on glial cell activation in wild-type mice *in vivo*, we found that pre- and posttreatment of sorafenib decreased the increases in Iba-1 immunofluorescence intensity, Iba-1-positive cell number, and percent of staining area induced by LPS (**Figure 3**, **6**). Pretreatment with sorafenib had similar effects on GFAP immunofluorescence intensity and percent of stained area (**Figure 4**). Additionally, posttreatment of sorafenib significantly suppressed GFAP immunofluorescence intensity, cell number, and percent of stained area in LPS-treated wild-type mice (**Figure 6**). How does pre- or posttreatment with sorafenib modulate the neuroinflammatory responses induced by LPS *in vivo*? Several studies have reported that multikinase inhibitors targeting VEGFRs and PDGFRs downregulate glial activation (8, 62). For instance, regorafenib suppresses the LPS-induced increases in Iba-1 and GFAP immunofluorescence intensity in wild-type mice (8), and in RIP-Tag2 mice, injection with VEGFRs and PDGFRβ shRNAs significantly decreases Iba-1 and GFAP immunointensity (62). Another VEGFRs and PDGFRs inhibitor, dabrafenib, significantly increases LPS-induced neuroinflammatory response-linked cell survival by inhibiting the hyperpermeability and leukocyte migration of blood cells in C57BL/6 mice (63). Given these previous observations, our findings suggest that pre- and posttreatment with sorafenib affects LPS-mediated microglial and astrocyte activation by inhibiting VEGFRs and PDGFRβ. Conversely, it is also possible that sorafenib posttreatment inhibits LPS-induced TLR4 activation to prevent VEGFRs and PDGFRβ signaling and alter LPS-mediated neuroinflammatory responses. Interestingly, we observed that pretreatment but not posttreatment of sorafenib altered the number of astrocytes in the hippocampus in wild-type mice (**Figures 4**, **6**). One possibility is that VEGFRs and PDGFRβ have limited involvement in astrocyte migration, and thus pretreatment of sorafenib has less of an effect on LPS-induced astrocytic neuroinflammation than posttreatment *in vivo*. In future work, we will further examine whether pre- and posttreatment of sorafenib alters LPS-evoked astrocytic neuroinflammatory responses in VEGFRs- and/or PDGFRβ-dependent manner in wild-type mice.

COX-2 is a typical proinflammatory marker and is released by activated microglia and astrocytes at the beginning of neuroinflammation (64). COX-2 expression supports the inflammatory process and has a significant role in cell proliferation, macrophage, and synoviocyte activation (2). Systemic exposure to LPS significantly increases COX-2 mRNA and protein expression in the hippocampus and cortex of mice (6, 7). In a mouse model of angiogenesis, injection of a VEGFR2 inhibitor, microRNA-101, diminishes COX-2 expression (65), and in human intestinal microvascular endothelial cells, curcumin reduces COX-2 mRNA levels by inhibiting VEGF (66). In addition, in rat smooth muscle cells, PDGF-induced expression of COX-2 is reduced by PDGFRβ inhibitors, and recombinant rat COX-2 cDNA is directly required for PDGFR-dependent stabilization of COX-2 mRNA, suggesting that PDGFR is required for regulating COX-2 expression (67). Importantly, we previously demonstrated that the VEGFRs and PDGFRβ inhibitor regorafenib significantly decreases the increase in COX-2 levels induced by LPS in wild-type mice (8). Consistent with these findings, sorafenib treatment reduced the LPS-induced increase in COX-2 levels in wild-type mice (**Figure 4**). It is possible that multikinase inhibitors like sorafenib and regorafenib regulate COX-2 expression *via* inhibition of VEGFR2 and PDGFRβ in response to neuroinflammation *in vivo*.

Studies in LPS-injected mouse models have shown that AKT-linked STAT3 signaling contributes to the regulation of proinflammatory responses in the brain (6, 7, 68). Xanthatin, a VEGFRs and PDGFRβ inhibitor, significantly reduces neuroinflammation by inhibiting AKT/PI3K/STAT3 signaling in a rat corneal alkali burn model (69). TKI258, another VEGFRs and PDGFRs inhibitor, reduces p-AKT and p-STAT3 levels in tumor xenograft nude mice (59). Similarly, we found that sorafenib pretreatment decreased p-AKT^S473^ and p-STAT3^S727^ levels in LPS-injected wild-type mice (**Figure 5**). Interestingly, sorafenib posttreatment reduced p-AKT^S473^ levels in LPS-injected wild-type mice but not p-STAT3 ^S727^ levels (**Figure 7**). Together, our findings and previous work suggest that sorafenib pre-or posttreatments differently regulate VEGFRs- and/or PDGFRβ-linked AKT and STAT3 signaling to modulate neuroinflammation in LPS-induced wild-type mice.

Neuroinflammation may impact learning and memory as well as synaptic function both directly and indirectly (70). Conflicting effects of sorafenib on cognitive/synaptic function have been reported. In APPswe mice (a mouse model of AD), sorafenib treatment modulates neuroinflammatory responses to restore working memory (15). On the contrary, negative effects of sorafenib on cognitive function *via* disruption of metabonomic pathways have been observed in cancer patients (71, 72). Therefore, here we examined whether sorafenib alters LPS-mediated pre- or postsynapse-linked proteins. In wild-type mice, sorafenib treatment significantly reversed the LPS-mediated alteration of shank-l fluorescence intensity but had no effects on synaptophysin and PSD-95 (**Supplementary Figure 3**). Thus, the present and previous findings indicate that sorafenib can regulate synaptic/cognitive function positively and/or negatively in LPS-treated mice and/or mouse models of AD. A limitation of this study is that mice were only i.p. treated with sorafenib daily for 3 days at a dose of 10 mg/kg. This may not have been a sufficient duration of treatment and/or dose to alter synaptic and cognitive function. The effects of longer treatment periods and/or higher doses of sorafenib on LPS-mediated synaptic and/or cognitive function will be assessed in future work.

Despite the availability of several models for evaluating the therapeutic potential of molecules (73, 74), the effects of sorafenib on neuroinflammation have rarely been studied in non-LPS models. To address this gap, we examined the effects of sorafenib on neuroinflammation in 5xFAD mice, a model of AD, which revealed that 3 consecutive days of treatments significantly reduced Aβ-mediated astroglial activation but not microglial activation (**Figure 8**). Further studies are needed to determine if longer treatment periods (i.e., daily for 2 weeks or 4 weeks) and/or higher doses of sorafenib are able to alter Aβ-induced microgliosis.

## Conclusion

**Figure 9:**
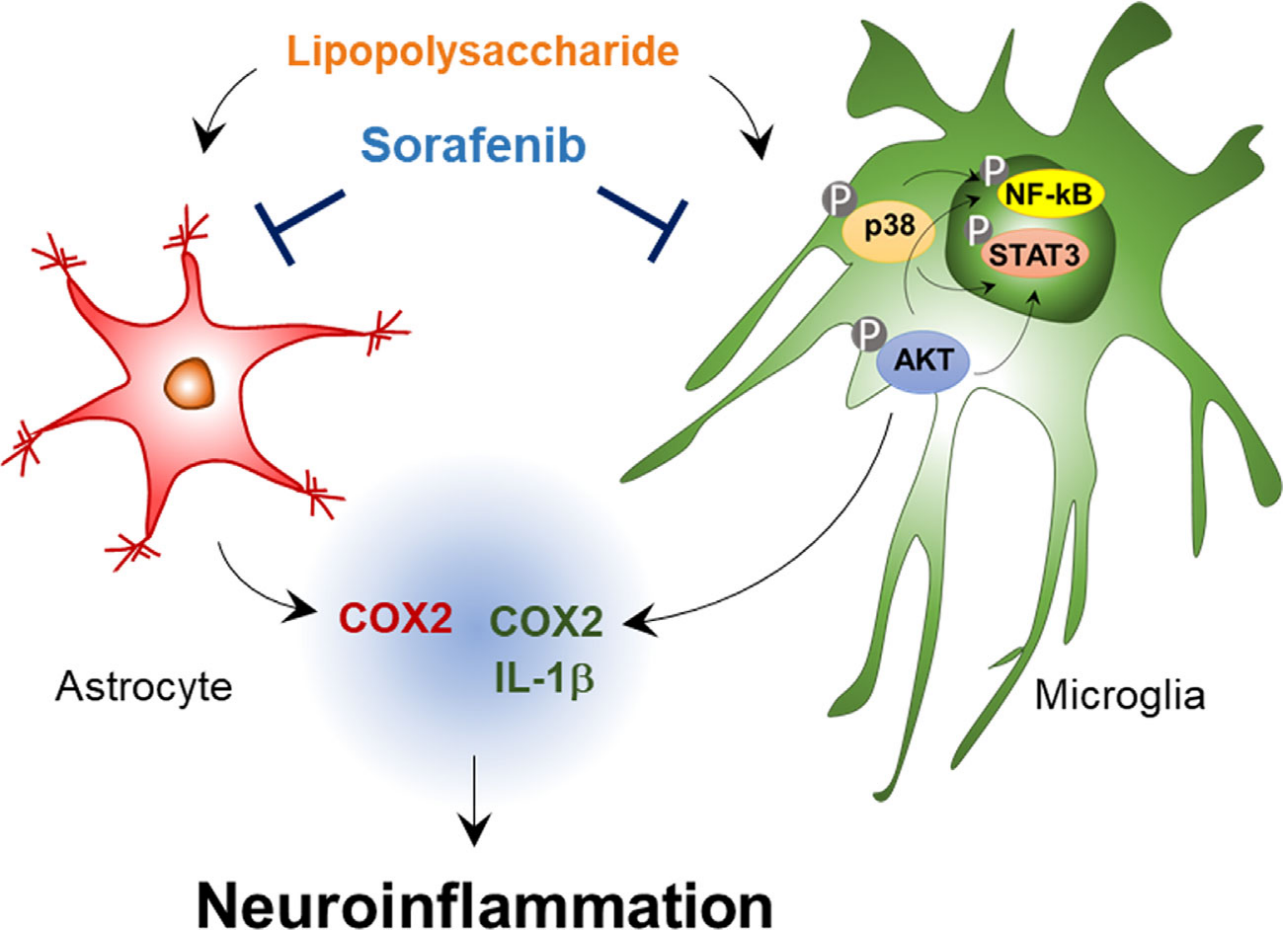
Sorafenib affects LPS-stimulated neuroinflammatory responses *in vitro* and *in vivo*. In BV2 microglial cells and wild-type mice, sorafenib reduces the effects of LPS on the mRNA levels of the proinflammatory cytokines IL-1β and COX-2 in microglia and COX-2 in astrocytes by modulating AKT/P38-associated NF-kB/STAT3 signaling pathways. Accordingly, sorafenib may have therapeutic potential for neuroinflammation-related diseases.

In summary, sorafenib, a multikinase inhibitor whose targets include VEGFR2 and PDGFRβ, reduces the effects of LPS on proinflammatory cytokine levels in BV2 cells and primary astrocytes (**Figure 9**). In addition, sorafenib suppresses the AKT/P38-linked STAT3/NF-kB signaling pathway, which plays a role in proinflammatory cytokine release, in BV2 cells. In wild-type mice, pre- and posttreatment of sorafenib significantly reduces the stimulation of microglial and astrocyte activation and COX-2 levels by LPS by inhibiting AKT signaling, and sorafenib suppresses Aβ-mediated astrogliosis but not microgliosis in a mouse model of AD. Thus, we suggest that sorafenib holds potential as a drug for protecting against acute neuroinflammation in the brain.

## Data Availability Statement

The original contributions presented in the study are included in the article/**Supplementary Material**. Further inquiries can be directed to the corresponding author.

## Ethics Statement

The animal study was reviewed and approved by Korea Brain research institute IACUC.

## Author Contributions

Study Conception and Design: JK and H-SH. Acquisition of data: JK, J-HP, and SKP. Preparation of figures: JK. Preparation of tables: JK. Writing of manuscript: JK and H-SH. All authors contributed to the article and approved the submitted version.

## Funding

This work was supported by the KBRI basic research program through KBRI funded by the Ministry of Science, ICT & Future Planning (grant numbers 21-BR-02-11, 21-BR-03-05, H-SH) and the National Research Foundation of Korea (grant number 2019R1A2B5B01070108, H-SH).

## Conflict of Interest

The authors declare that the research was conducted in the absence of any commercial or financial relationships that could be construed as a potential conflict of interest.
