# Supplementary material for: Sorafenib Modulates the LPS- and Aβ-Induced Neuroinflammatory Response in Cells, Wild-Type Mice, and 5xFAD Mice
Source: Front Immunol. 2021 May 27;12:684344. doi: 10.3389/fimmu.2021.684344 (PMC8190398; doi:10.3389/fimmu.2021.684344)
Supplement: Supplementary file 1 [file DataSheet_1.pdf]

Supplementary information

Sorafenib modulates the LPS- and Aβ-induced neuroinflammatory response in cells, wild-type mice, and 5xFAD mice

Jieun Kim[1], Jin-Hee Park[1], Seon Kyeong Park[1], Hyang-Sook Hoe[1,2*]

[1]Department of Neural Development and Disease, Korea Brain Research Institute (KBRI), 61, Cheomdan-ro, Dong-gu, Daegu, Korea. 41068; [2]Department of Brain & Cognitive Sciences, Daegu Gyeongbuk Institute of Science & Technology (DGIST), Daegu, Korea, 42988

*Corresponding author

Hyang-Sook Hoe, Ph.D.: Department of Neural Development and Disease, Korea Brain Research Institute (KBRI), 61 Cheomdan-ro, Dong-gu, Daegu, Korea, 41068

E-mail: sookhoe72@kbri.re.kr

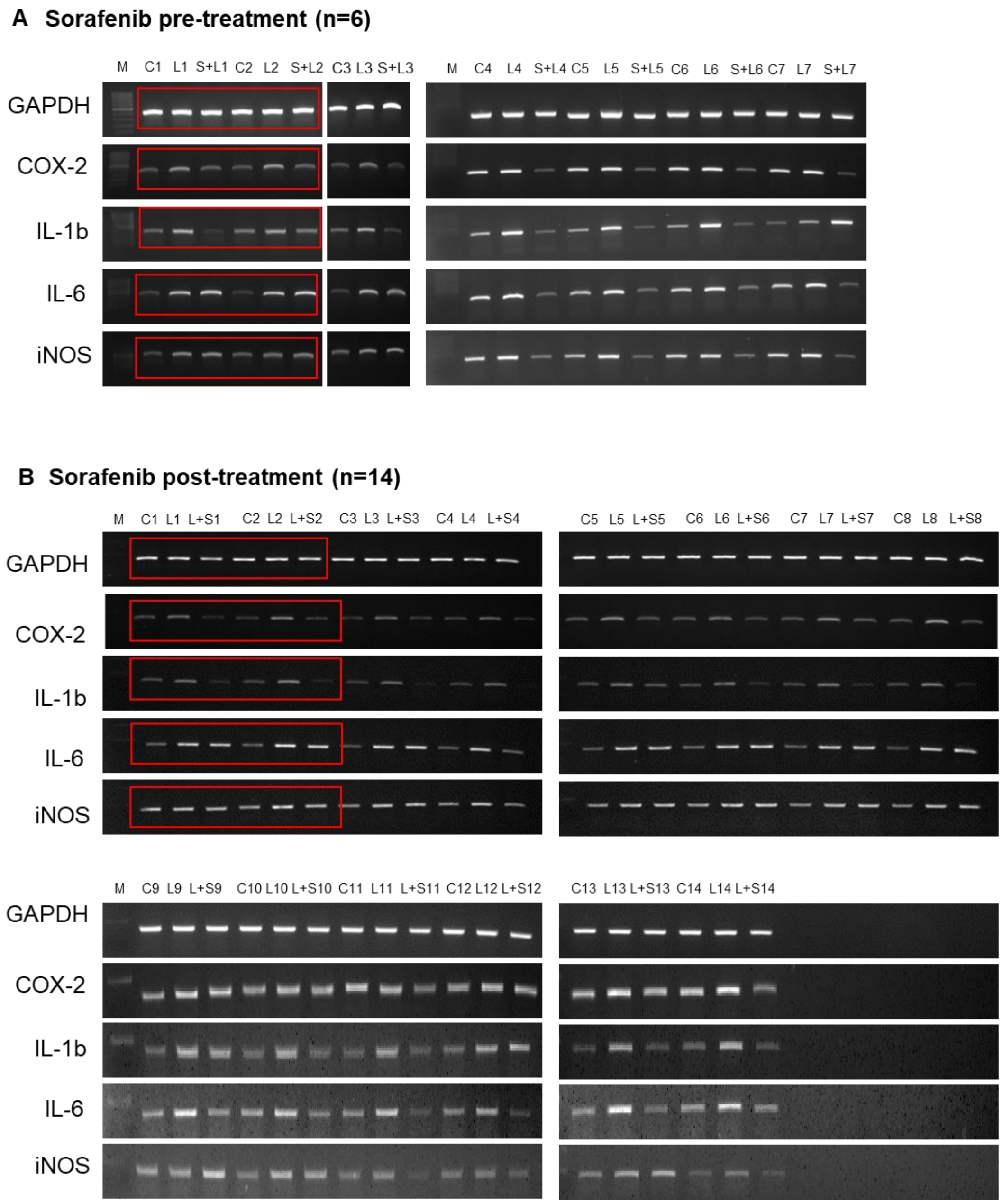

Supplementary Figure 1. Original raw data from RT-PCR. (A) Raw data from RT-PCR analysis of proinflammatory cytokine levels in BV2 cells pretreated with sorafenib (n=7/group). (B) Raw data from RT-PCR analysis of proinflammatory cytokine levels in BV2 cells posttreated with sorafenib (n=14/group).

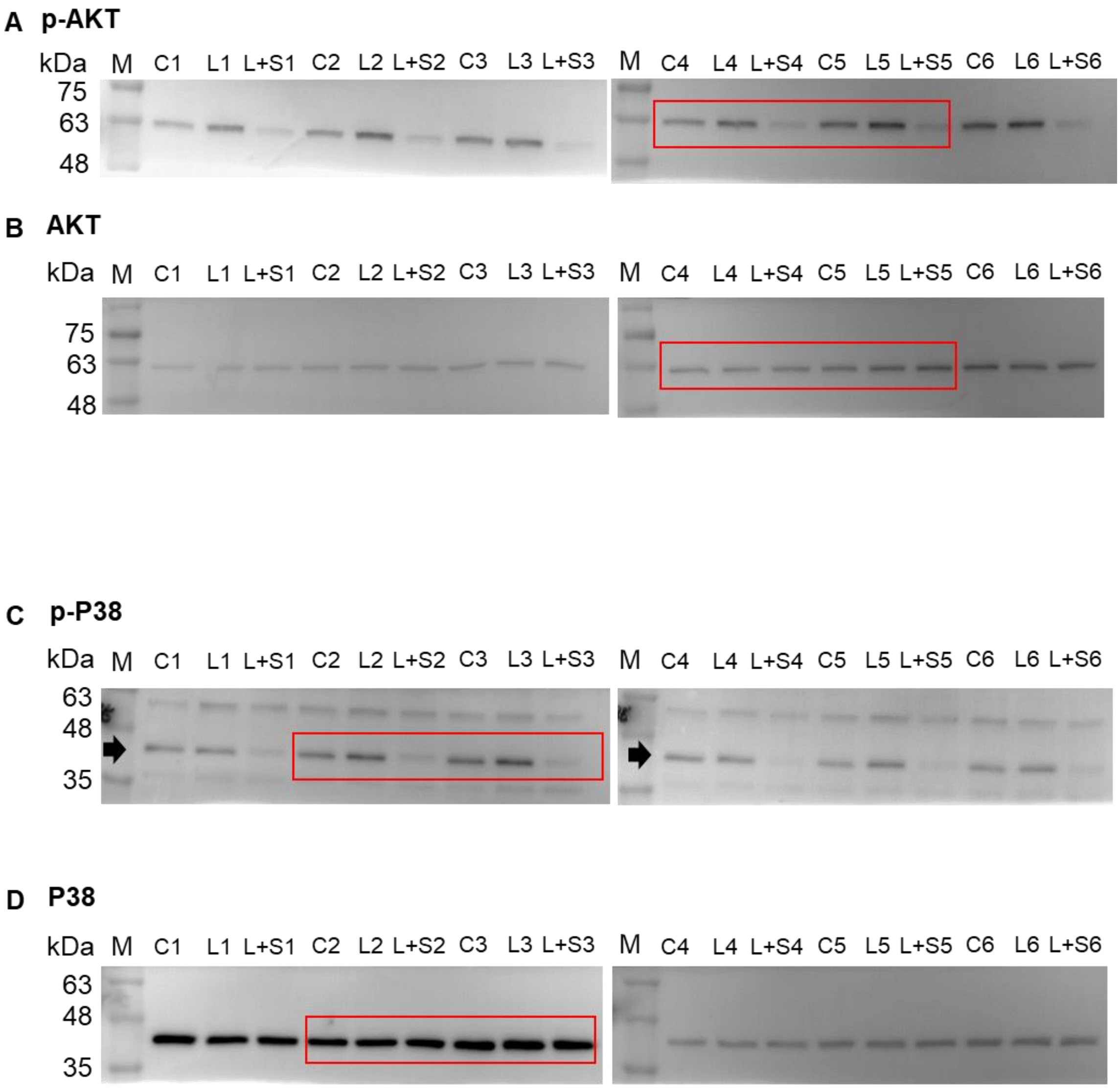

Supplementary Figure 2. Original raw data from Western blotting. (A) Raw data from Western blot analysis of p-AKT and AKT levels in BV2 cells posttreated with sorafenib (n=6/group). (B) Raw data from Western blot analysis of P-p38 and p38 levels in BV2 cells posttreated with sorafenib (n=6/group).

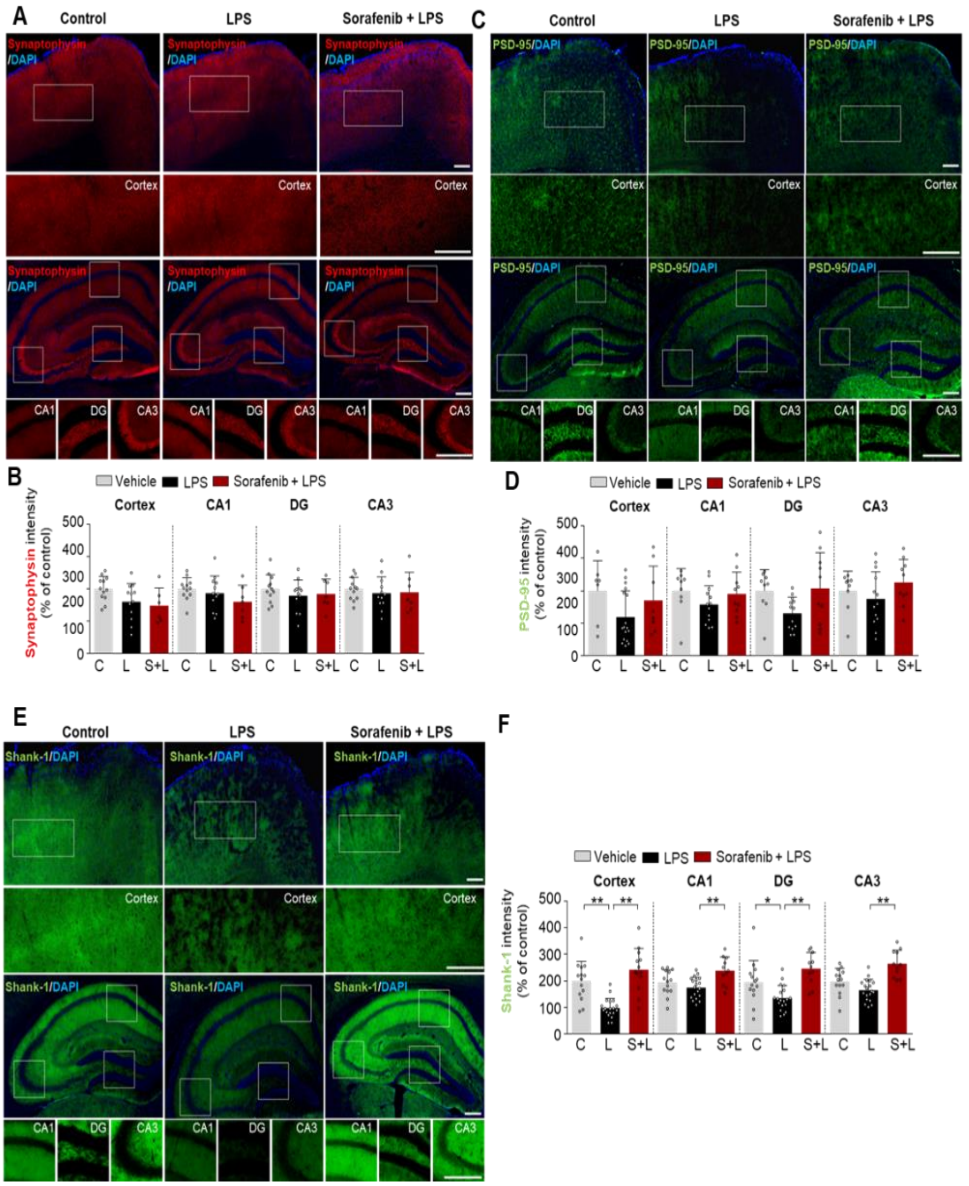

Supplementary Figure 3. Sorafenib upregulated the LPS-mediated decrease in shank-1 intensity in wild-type mice. (**A, C, E**) Immunofluorescence staining with anti-synaptophysin, anti-PSD-95, and anti-shank-1 antibodies of brain slices from wild-type mice pretreated with sorafenib followed by LPS. (**B, D, F**) Quantification of the data in **A**, **C**, and **E** (analyzed number of brain slices/images (n), B: Vehicle, n=12 ; LPS, n=12; Sorafenib + LPS, n=6, D: Vehicle, n=8 ; LPS, n=13; Sorafenib + LPS, n=8, F: Vehicle, n=14 ; LPS, n=19; Sorafenib + LPS, n=13). Scale bar = 200 µM. $*p < 0.05$, $**p < 0.01$.

Materials and Methods

Supplementary Table 1. List of antibodies used in supplementary figures in this study

**Primary antibodies**					
**Antigen**	**Host species**	**Dilution**	**Manufacturer**	**Catalog no.**	**Analysis**
Iba-1	Rabbit polyclonal	1:500	Wako	019-19741	IHC
GFAP	Rabbit polyclonal	1:500	Neuromics	RA22101	IHC
Synaptophysin	Rabbit polyclonal	1:200	Sigma	S5768	IHC
PSD95	Mouse monoclonal	1:200	Neuromap	75028	IHC
Shank-1	Mouse monoclonal	1:200	Santa Cruz	SC393963	IHC
**Secondary antibodies**					
**Antibody**		**Dilution**	**Manufacturer**	**Catalog no.**	**Analysis**
Goat anti-rabbit IgG, Alexa Fluor 488		1:200	Invitrogen	A11008	IHC
Goat anti-mouse IgG, Alexa Fluor 555		1:500	Invitrogen	A21422	IHC
Goat anti-mouse IgG, Alexa Fluor 488		1:200	Invitrogen	A11001	ICC

Supplementary Table 2. Results of the Kolmogorov-Smirnov or Shapiro-Wilk nomality test and significance of the results of the *in vitro* experiments in this study.

**Figure 1B MTT 6 h**	**0.1 Veh**	**0.1 uM**	**1 Veh**	**1 uM**	**5 Veh**	**5 uM**	**10 Veh**	**10 uM**	**20 Veh**	**20 uM**
KS distance	0.1815	0.2469	0.264	0.2391	0.1741	0.2168	0.1495	0.2984	0.3103	0.2896
P value	>0.1000	>0.1000	>0.1000	>0.1000	>0.1000	>0.1000	>0.1000	>0.1000	0.0729	>0.1000
Passed normality test (alpha=0.05)?	Yes	Yes	Yes	Yes	Yes	Yes	Yes	Yes	Yes	Yes
**Figure 1C MTT 24 h**	**0.1 Veh**	**0.1 uM**	**1 Veh**	**1 uM**	**5 Veh**	**5 uM**	**10 Veh**	**10 uM**	**25 Veh**	**25 uM**
KS distance	0.1606	0.2755	0.2005	0.3043	0.225	0.2368	0.3182	0.3116	0.3315	0.3228
P value	>0.1000	>0.1000	>0.1000	>0.1000	>0.1000	>0.1000	>0.1000	>0.1000	0.0760	0.0965
Passed normality test (alpha=0.05)?	Yes	Yes	Yes	Yes	Yes	Yes	Yes	Yes	Yes	Yes
**Figure 1F COX-2**				**Control**			**LPS**			**LPS + Sorafenib**
KS normality test, KS distance				0.1102			0.2905			0.2885
P value				>0.1000			0.0758			0.0806
Passed normality test (alpha=0.05)?				Yes			Yes			Yes
**Figure 1F IL-1β**										
KS distance				0.2161			0.2653			0.3944
P value				>0.1000			>0.1000			0.0015
Passed normality test (alpha=0.05)?				Yes			Yes			No
**Figure 1F IL-6**										
KS distance				0.2309			0.297			0.2272
P value				>0.1000			0.0619			>0.1000
Passed normality test (alpha=0.05)?				Yes			Yes			Yes
**Figure 1F iNOS**										
KS distance				0.2077			0.2396			0.2963
P value				>0.1000			>0.1000			0.0634
Passed normality test (alpha=0.05)?				Yes			Yes			Yes
**Figure 1I COX-2**				**Control**			**LPS**			**Sorafenib + LPS**
KS distance				0.1105			0.1194			0.1277
P value				>0.1000			>0.1000			>0.1000
Passed normality test (alpha=0.05)?				Yes			Yes			Yes
**Figure 1I IL-1β**										
KS distance				0.1053			0.1354			0.1639
P value				>0.1000			>0.1000			>0.1000
Passed normality test (alpha=0.05)?				Yes			Yes			Yes
**Figure 1I IL-6**										
KS distance				0.1495			0.1045			0.2024
P value				>0.1000			>0.1000			>0.1000
Passed normality test (alpha=0.05)?				Yes			Yes			Yes
**Figure 1I iNOS**										
KS distance				0.1102			0.2905			0.2885
P value				>0.1000			0.0758			0.0806
Passed normality test (alpha=0.05)?				Yes			Yes			Yes
**Figure 1J COX-2**										
Shapiro-Wilk normality test,W				0.8437			0.9954			0.9355
P value				0.2064			0.9831			0.6272
Passed normality test (alpha=0.05)?				Yes			Yes			Yes
**Figure 1J IL-1β**										
W				0.9885			0.7226			0.7766
P value				0.9498			0.0207			0.0665
Passed normality test (alpha=0.05)?				Yes			No			Yes
**Figure 1J IL-6**										
W				0.9581			0.8025			0.7526
P value				0.7670			0.1068			0.0408
Passed normality test (alpha=0.05)?				Yes			Yes			No

**Figure 1J iNOS**			
W	0.8655	0.97	0.8598
P value	0.2804	0.8416	0.2595
Passed normality test (alpha=0.05)?	Yes	Yes	Yes
**Figure 2B p-AKT^{s473}**			
KS distance	0.1962	0.2911	0.1987
P value	>0.1000	>0.1000	>0.1000
Passed normality test (alpha=0.05)?	Yes	Yes	Yes
**Figure 2B AKT**			
KS distance	0.2062	0.1542	0.1529
P value	>0.1000	>0.1000	>0.1000
Passed normality test (alpha=0.05)?	Yes	Yes	Yes
**Figure 2C p-P38$^{T180/Y182}$**			
KS distance	0.1843	0.1504	0.1659
P value	>0.1000	>0.1000	>0.1000
Passed normality test (alpha=0.05)?	Yes	Yes	Yes
**Figure 2C P38**			
KS distance	0.2359	0.1972	0.1726
P value	>0.1000	>0.1000	>0.1000
Passed normality test (alpha=0.05)?	Yes	Yes	Yes
**Figure 2E p-STAT3^{s727}**			
KS distance	0.09941	0.06524	0.06516
P value	<0.0001	0.0009	0.0009
Passed normality test (alpha=0.05)?	No	No	No
**Figure 2F p-NF-kB^{s536}**			
KS distance	0.09698	0.114	0.105
P value	<0.0001	<0.0001	<0.0001
Passed normality test (alpha=0.05)?	No	No	No

Supplementary Table 3. Results of the Kolmogorov-Smirnov or Shapiro-Wilk nomality test and significance of the results of the *in vivo* experiments in this study.

**Brain regions**		**Cortex**			**CA1**			**DG**			**CA3**	
**Figure 3B**	**Vehicle**	**LPS**	**Sorafenib + LPS**	**Vehicle**	**LPS**	**Sorafenib + LPS**	**Vehicle**	**LPS**	**Sorafenib + LPS**	**Vehicle**	**LPS**	**Sorafenib + LPS**
KS normality test, KS distance	0.1185	0.1141	0.1493	0.1279	0.1617	0.1626	0.0961	0.1408	0.0952	0.1281	0.1623	0.108
P value	>0.1000	>0.1000	>0.1000	>0.1000	>0.1000	>0.1000	>0.1000	>0.1000	>0.1000	>0.1000	>0.1000	>0.1000
Passed normality test (alpha=0.05)?	Yes	Yes	Yes	Yes	Yes	Yes	Yes	Yes	Yes	Yes	Yes	Yes
**Figure 3D**												
KS distance	0.1443	0.1565	0.2011	0.2464	0.118	0.1831	0.3432	0.1786	0.1962	0.3432	0.1786	0.1962
P value	>0.1000	>0.1000	0.0660	0.0051	>0.1000	0.0935	<0.0001	>0.1000	0.0525	<0.0001	>0.1000	0.0525
Passed normality test (alpha=0.05)?	Yes	Yes	Yes	No	Yes	Yes	No	Yes	Yes	No	Yes	Yes
**Figure 4B**												
KS distance	0.2517	0.196	0.1307	0.1636	0.148	0.1911	0.2138	0.1874	0.1368	0.1639	0.2442	0.1161
P value	0.0078	0.0658	>0.1000	>0.1000	>0.1000	>0.1000	0.0635	0.0776	>0.1000	>0.1000	0.0041	>0.1000
Passed normality test (alpha=0.05)?	No	Yes	Yes	Yes	Yes	Yes	Yes	Yes	Yes	Yes	No	Yes
**Figure 4C**												
KS distance	0.1339	0.2277	0.09013	0.1316	0.1955	0.1595	0.1636	0.1969	0.1245	0.116	0.1067	0.1383
P value	>0.1000	0.0144	>0.1000	>0.1000	0.0543	>0.1000	>0.1000	0.0509	>0.1000	>0.1000	>0.1000	>0.1000
Passed normality test (alpha=0.05)?	Yes	No	Yes	Yes	Yes	Yes	Yes	Yes	Yes	Yes	Yes	Yes
**Figure 5B**												
KS distance	0.2845	0.2904	0.2384	0.3419	0.127	0.1751	0.2116	0.2018	0.2555	0.1231	0.2126	0.2708
P value	0.0012	0.0008	0.0417	<0.0001	>0.1000	>0.1000	0.0322	>0.1000	0.0137	>0.1000	0.0399	0.0152
Passed normality test (alpha=0.05)?	No	No	No	No	Yes	Yes	No	Yes	No	Yes	No	No
**Figure 5D**												
KS distance	0.1538	0.2483	0.241	0.08947	0.216	0.2195	0.1643	0.2297	0.3169	0.1083	0.1836	0.2354
P value	>0.1000	0.0565	0.0268	>0.1000	0.0200	0.0288	>0.1000	0.0095	<0.0001	>0.1000	0.0916	0.0131
Passed normality test (alpha=0.05)?	Yes	Yes	No	Yes	No	No	Yes	No	No	Yes	Yes	No
**Figure 6C**	**Vehicle**	**LPS**	**LPS + Sorafenib**	**Vehicle**	**LPS**	**LPS + Sorafenib**	**Vehicle**	**LPS**	**LPS + Sorafenib**	**Vehicle**	**LPS**	**LPS + Sorafenib**
KS distance	0.1421	0.132	0.2122	0.118	0.2672	0.1103	0.1493	0.1723	0.1812	0.1628	0.1212	0.1529
P value	>0.1000	>0.1000	0.0144	>0.1000	0.0014	>0.1000	>0.1000	>0.1000	0.0841	>0.1000	>0.1000	>0.1000
Passed normality test (alpha=0.05)?	Yes	Yes	No	Yes	No	Yes	Yes	Yes	Yes	Yes	Yes	Yes
**Figure 6E**												
KS distance	0.2254	0.2175	0.1406	0.2254	0.2175	0.1406	0.1376	0.08628	0.1476	0.1449	0.1548	0.09275
P value	0.0037	0.0081	>0.1000	0.0037	0.0081	>0.1000	>0.1000	>0.1000	>0.1000	>0.1000	>0.1000	>0.1000
Passed normality test (alpha=0.05)?	No	No	Yes	No	No	Yes	Yes	Yes	Yes	Yes	Yes	Yes
**Figure 7B**												
KS distance	0.1685	0.1151	0.1145	0.2435	0.137	0.1222	0.149	0.152	0.1272	0.1198	0.1291	0.1439
P value	>0.1000	>0.1000	>0.1000	0.0239	>0.1000	>0.1000	>0.1000	>0.1000	>0.1000	>0.1000	>0.1000	>0.1000
Passed normality test (alpha=0.05)?	Yes	Yes	Yes	No	Yes	Yes	Yes	Yes	Yes	Yes	Yes	Yes
**Figure 7E**												
KS distance	0.1982	0.2359	0.274	0.1486	0.165	0.2164	0.1814	0.2428	0.3191	0.1099	0.2034	0.2953
P value	>0.1000	0.0336	0.0004	>0.1000	>0.1000	0.0195	>0.1000	0.0175	<0.0001	>0.1000	0.0957	<0.0001
Passed normality test (alpha=0.05)?	Yes	No	No	Yes	Yes	No	Yes	No	No	Yes	Yes	No

**Brain regions**	**Cortex**		**CA1**		**DG**		**CA3**	
**Figure 8C**	**Vehicle**	**Sorafenib**	**Vehicle**	**Sorafenib**	**Vehicle**	**Sorafenib**	**Vehicle**	**Sorafenib**
KS distance	0.2106	0.1299	0.2302	0.1192	0.1676	0.2015	0.1284	0.102
P value	0.0338	>0.1000	0.0092	>0.1000	>0.1000	0.0325	>0.1000	>0.1000
Passed normality test (alpha=0.05)?	No	Yes	No	Yes	Yes	No	Yes	Yes
**Figure 8E**								
KS distance	0.1299	0.1088	0.1451	0.1366	0.2025	0.162	0.2107	0.1213
P value	>0.1000	>0.1000	>0.1000	>0.1000	0.0493	>0.1000	0.0337	>0.1000
Passed normality test (alpha=0.05)?	Yes	Yes	Yes	Yes	No	Yes	No	Yes

Brain regions	Cortex			CA1			DG			CA3		
**Supplementary Figure 3B**	**Vehicle**	**LPS**	**Sorafenib + LPS**	**Vehicle**	**LPS**	**Sorafenib + LPS**	**Vehicle**	**LPS**	**Sorafenib + LPS**	**Vehicle**	**LPS**	**Sorafenib + LPS**
KS distance	0.1519	0.1714	0.231	0.131	0.1293	0.1257	0.1154	0.1759	0.2076	0.1256	0.1445	0.2163
P value	>0.1000	>0.1000	>0.1000	>0.1000	>0.1000	>0.1000	>0.1000	>0.1000	>0.1000	>0.1000	>0.1000	>0.1000
Passed normality test (alpha=0.05)?	Yes	Yes	Yes	Yes	Yes	Yes	Yes	Yes	Yes	Yes	Yes	Yes
**Supplementary Figure 3D**												
KS distance	0.2946	0.2401	0.2488	0.2192	0.1068	0.1674	0.2737	0.1715	0.1663	0.2709	0.1125	0.1803
P value	0.0668	0.0102	>0.1000	>0.1000	>0.1000	>0.1000	0.0506	>0.1000	>0.1000	0.0557	>0.1000	>0.1000
Passed normality test (alpha=0.05)?	Yes	No	Yes	Yes	Yes	Yes	Yes	Yes	Yes	Yes	Yes	Yes
**Supplementary Figure 3F**												
KS distance	0.144	0.1514	0.1432	0.1526	0.1074	0.1261	0.1418	0.1805	0.1202	0.1648	0.07327	0.1636
P value	>0.1000	>0.1000	>0.1000	>0.1000	>0.1000	>0.1000	>0.1000	>0.1000	>0.1000	>0.1000	>0.1000	>0.1000
Passed normality test (alpha=0.05)?	Yes	Yes	Yes	Yes	Yes	Yes	Yes	Yes	Yes	Yes	Yes	Yes
**Supplementary Figure 3H**	**Vehicle**	**Sorafenib**										
KS distance	0.1051	0.119										
P value	>0.1000	>0.1000										
Passed normality test (alpha=0.05)?	Yes	Yes										
